# Optimization of the Extraction of Bioactive Compounds from Walnut (*Juglans major 209 x Juglans regia)* Leaves: Antioxidant Capacity and Phenolic Profile

**DOI:** 10.3390/antiox9010018

**Published:** 2019-12-24

**Authors:** Adela Fernández-Agulló, Aída Castro-Iglesias, María Sonia Freire, Julia González-Álvarez

**Affiliations:** Department of Chemical Engineering, School of Engineering, Universidade de Santiago de Compostela, 15782 Santiago de Compostela, Spain; adela.fernandez@usc.es (A.F.-A.); aida_9790@hotmail.com (A.C.-I.); mariasonia.freire@usc.es (M.S.F.)

**Keywords:** walnut leaves, *Juglans major 209 x Juglans regia*, maceration, phenolic compounds, antioxidant activity, UPLC/ESI-QTOF-MS, response surface methodology

## Abstract

This work studies the extraction of phenolic compounds from walnut leaves of the hybrid *Juglans major 209 x Juglans regia* based on extract antioxidant capacity. Once the solid/liquid ratio was selected (1/10 g/mL), by means of a Box-Benkhen experimental design, the influence of temperature (25–75 °C), time (30–120 min), and aqueous ethanol concentration (10–90%) on extraction yield and ferric reducing antioxidant power (FRAP), 2,2-diphenyl-1-picrylhydrazyl (DPPH), and 2,2’-azinobis-3-ethylbenzothiazoline-6-sulfonic acid (ABTS) antioxidant activities were analyzed. In all cases, the quadratic effect of % EtOH was the most significant, followed by the linear effect of temperature and, for most of the responses, the effect of time was almost negligible. Response surface analysis allowed to select the optimal extraction conditions: 75 °C, 120 min and 50% ethanol, which led to the following extract properties: extraction yield, 30.17%; FRAP, 1468 nmol ascorbic acid equivalents (AAE)/mg extract d.b.; DPPH, 1.318 mmol Trolox equivalents (TRE)/g extract d.b.; DPPH EC_50_, 0.11 mg/mL; ABTS, 1.256 mmol TRE/g extract (on dry basis) and ABTS EC_50_, 0.985 mg/mL. Quercetin 3-*β*-D-glucoside, neochlorogenic acid, and chlorogenic acid, in this order, were the main compounds identified in this extract by ultra-performance liquid chromatography coupled with electrospray ionization and time-of-flight mass spectrometry (UPLC/ESI-QTOF-MS), with various potential applications that support this valorization alternative for walnut leaves.

## 1. Introduction

*Juglans major 209 x Juglans regia* is a walnut hybrid species intended to produce good-quality wood. Although wood is the main product, the use of other fractions that can be considered as wastes, such as the leaves, would contribute to more profitable production and to a more sustainable plantation management. 

Walnut leaves have been intensively used in traditional medicine, and various studies have demonstrated the antimicrobial and antioxidant properties of the extracts from the leaves of several walnut (*Juglans regia)* cultivars [1,2,3,4]. Carvalho et al. [3] also demonstrated the antihemolytic and human renal cancer cell antiproliferative activities of walnut leaf methanolic extracts. Quantitative and qualitative determinations of the phenolic compounds present in walnut (*Juglans regia*) leaves have also been carried out [1,4,5,6] demonstrating quite significant variations in the extract composition. Amaral et al. [5] identified seven phenolic compounds in methanol and acidified water extracts of walnut leaves, being quercetin 3-*O*-galactoside the major compound while *4*-*p*-*coumaroylquinic* acid was the minor one. 3-*O*-caffeoylquinic acids and quercetin *O*-pentoside were the main phenolic compounds among the 25 phenolic compounds identified in methanol and decoction extracts by Santos et al. [4]. Pereira et al. [1] identified 10 compounds in aqueous extracts: 3- and 5-caffeoylquinic acids, 3- and 4-*p*-coumaroylquinic acids, *p*-coumaric acid, quercetin 3-*O*-galactoside, quercetin 3-*O*-pentoside derivative, quercetin 3-*O*-arabinoside, quercetin 3-*O*-xyloside and quercetin 3-*O*-rhamnoside. In addition to phenolic acids and flavonoids, Nour et al. [6] also reported the presence of juglone (5-hydroxy-1,4-napthoquinone) in walnut leaves, ellagic acid as the dominating phenolic acid and myricetin, catechin hydrate and rutin as the main flavonoids. 

Different solvents have been used for the extraction of phenolic compounds from walnut leaves: Aqueous ethanol [2,7], methanol [4,5], methanol with 1% butylated hydroxytoluene (BHT) [6], water [1,4], acidified water [5], and chloroform [5]. In this work, looking for a green extraction process, extraction was performed by maceration with aqueous ethanol, a bio-solvent produced from biomass, completely biodegradable [8], and generally recognized as safe (GRAS) solvent. In addition to the type of solvent, other factors that can affect the efficiency of the extraction process that were examined in this work are the extraction temperature, the extraction time, and the solid to solvent ratio [9]. 

The aim of this work was to provide a means for the disposal and valorization of walnut leaves based on extract antioxidant activity, giving the walnut plantation an additional value to that provided by wood. As far as we know, this is the first work on the extraction and characterization of phenolic compounds of walnut leaves of the hybrid *Juglans major 209 x Juglans regia.* An experimental design combined with response surface methodology was used to optimize the extraction of phenolic compounds from walnut leaves based on extract antioxidant activity. The phytochemical characterization of the extracts was carried out and the results compared with those previously obtained for leaves from the *Juglans regia* species. 

## 2. Materials and Methods

### 2.1. Reagents and Standards

Acetic acid, sodium acetate, FeCl_3_·6H_2_O, HCl, l-ascorbic acid, methanol, ethanol, and potassium persulfate were purchased from Panreac (Barcelona, Spain). 2,4,6-Tripyridyl-s-triazine (TPTZ), Trolox (6-hydroxy-2,5,7,8-tetramethylchroman-2-carboxylic acid) and DPPH (2,2-diphenyl-1-picrylhydrazyl) were purchased from Fluka (Steinheim, Germany). ABTS (2,2’-azinobis-3-ethylbenzothiazoline-6-sulfonic acid) and HPLC standards: (–)-epigallocatechin, (–)-gallocatechin, catechin hydrate, chlorogenic acid, ellagic acid, epicatechin, ferulic acid, gallic acid, isorharmnetin, kaempferol, neochlorogenic acid, *p*-coumaric acid, procyanidin B2, quercetin, quercetin 3-*β*-D glucoside, and taxifolin, were obtained from Sigma (Steinheim, Germany).

### 2.2. Materials

Walnut (*Juglans major 209 x Juglans regia*) leaves were collected in July 2018 in a plantation located in A Coruña, Spain, dried at room temperature, milled, sieved, and the fraction of particle size between 0.1 and 1 mm was selected and stored protected from light till analysis. 

### 2.3. Extraction

Walnut leaves were extracted with aqueous ethanol in an orbital shaker (UNITRONIC-OR, Selecta (Barcelona, Spain) with temperature control at a shaking speed of 90 rpm. The solid/liquid ratio, temperature, time, and EtOH concentration were fixed for each experiment according to the established experimental planning (Table 1 and Table 2). The extract was separated by vacuum filtration, concentrated in a Büchi R-210 rotavapor and finally dried under vacuum to obtain a dry powder. Extraction yield was determined as the weight loss percentage of the initial walnut leaves.

The choice of the independent variables to be analyzed and their respective variation intervals was based on previous investigations on the extraction of phenolic compounds from various lignocellulosic materials using aqueous alcohols [10,11,12]. In the first stage, the influence of the solid/liquid ratio (1/5, 1/7.5 and 1/10 g/mL) was analyzed for fixed values of the other variables: Temperature, 50 °C, time, 60 min, and ethanol concentration, 50% (Table 1). Extraction yield and extract ferric reducing antioxidant power (FRAP) antioxidant activity were determined. All the assays were replicated, and the results expressed as mean value and standard deviation. The existence of significant differences among the results depending on the solid/liquid ratio used was analyzed by applying one-way ANOVA together with the Tukey’s test at a confidence level of 95% using the IBM SPSS Statistics 24 software (New York, NY, USA). 

Once S/L ratio was fixed at 1/10, a Box-Behnken experimental design was applied to analyze the influence of temperature (*x*_1_; 25, 50, and 75 °C), time (*x*_2_; 30, 75, and 120 min) and aqueous ethanol concentration (*x*_3_; 10%, 50%, and 90%) on extraction yield (*Y*_1_, g extract/100 g leaves on dry basis (d.b.)) and extract antioxidant activity determined according to the FRAP (*Y*_2_, nmol AAE/mg extract d.b.), DPPH (*Y*_3_, mmol TRE/g extract d.b.), and ABTS (*Y*_4_, mmol TRE/g extract d.b.) assays. The phenolic profile of the extract selected as the optimum was analyzed by ultra-performance liquid chromatography coupled with electrospray ionization and time-of-flight mass spectrometry (UPLC/ESI-QTOF-MS). 

### 2.4. Box-Behnken Experimental Design

The Box-Behnken experimental design applied consisted of 12 replicated experiments and 2 replicates in the central point (Table 2). The experiments were randomized to avoid unpredictable effects on the responses. Experimental results were analyzed using the IBM SPSS Statistics 24 software and fitted to polynomials of the form:

(1) $$Y\;=\;a_{0}+\;\overset{3}{\underset{i\;=\;0}{\sum }}a_{i}\;x_{i}^{*}+\overset{2}{\underset{i\;=\;1}{\sum }}\overset{3}{\underset{\begin{array}{c} j\;=\;2\\ j\;>\;i \end{array}}{\sum }}a_{ij}x_{i}^{*}x_{j}^{*}+\;\overset{3}{\underset{i\;=\;1}{\sum }}a_{ii}\;x_{i}^{*2}$$

where *Y* is the dependent variable or response, *a*_0_ is a scaling constant, *a_i_* represents the linear coefficients, *a_ij_* the interaction coefficients, *a_ii_* the quadratic coefficients, and $x_{i}^{*}$ the independent variables coded at three levels: −1 (lower limit), 0 (central point), and +1 (upper limit) (Table 2). Analysis of variance (ANOVA) was applied to determine the validity of the quadratic model as well as the statistical significance of the regression coefficients at a 95% confidence level. Moreover, to confirm the model’s accuracy, predicted values for each dependent variable were calculated and compared with the experimental ones (Table 2). The equations obtained for each dependent variable were visualized as response surface plots. 

### 2.5. Analytical Techniques

In order to have a more complete characterization of the antioxidant activity of the extracts, various methods were used, namely, the DPPH and ABTS assays based on the capacity to scavenge free radicals and the FRAP assay that measures the capacity to reduce a metal ion. 

The DPPH radical scavenging ability of the extracts was determined following the method proposed by Barreira et al. [13] modified as described in Vázquez et al. [11]. The results were expressed as mmol Trolox equivalent (TRE) per g extract d.b. and as the EC_50_ value, or extract concentration necessary to achieve a 50% DPPH radical inhibition. ABTS scavenging activity was determined according to the method of Re et al. [14], and the results expressed as mmol Trolox equivalent (TRE) per g extract d.b. and as the EC_50_ value. The FRAP assay was done according to Szöllösi and Szöllösi-Varga [15]. The results were expressed as nmol ascorbic acid equivalent (AAE) per mg extract d.b.

Phenolic compounds in the extract selected as the optimum were determined by UPLC/ESI-QTOF-MS using a Bruker Elute UHPLC (Billerica, MA, USA) and a Bruker TimsTOF (Billerica, MA, USA). Separations were performed using a Bruker Intensity Solo C18 2 µm (2.1 mm × 100 mm) column (Billerica, MA, USA) and a binary gradient of 0.1% aqueous formic acid for mobile phase A and 0.1% formic acid in methanol for mobile phase B at a flow rate of 0.25 mL/min. The LC gradient was 5% B from 0 to 0.4 min, from 5% to 35% B from 0.4 to 0.5 min, from 35% to 100% B from 0.5 to 7 min, 100% B from 7 to 12 min, from 100% to 5% B from 12 to 12.1 min and 5% B from 12.1 to 15 min. Table 3 shows the regression equation for each standard compound analyzed. As observed, all the compounds showed good linearity in a relatively wide concentration range.

## 3. Results and Discussion

### 3.1. Effect of the Solid/Liquid Ratio on Extraction Yield and Extract Antioxidant Activity

The influence of the solvent to walnut leaves ratio on the extraction yield and extract FRAP antioxidant activity is shown in Table 1. The extraction yield was positively influenced by decreasing S/L from 1/5 to 1/7.5. However, a further decrease to 1/10 didn’t improve the extraction yield significantly. On the other hand, the extract FRAP antioxidant activity increased with decreasing the S/L ratio in the whole range analyzed. These results were in agreement with previous research on the extraction of phenolic compounds from various plant materials [7,16,17] and can be explained by analyzing the concentration gradient of phenolics between walnut leaves and the solvent. The lower the S/L ratio, the greater the concentration gradient and consequently the extraction rate. Therefore, a S/L ratio of 1/10 was selected for subsequent experiments on analyzing the impact of temperature, time, and ethanol concentration on the extraction of phenolic compounds from walnut leaves. A greater decrease in the S/L ratio was not considered since it would mean not only a higher solvent consumption but also a higher energy cost in the extraction and solvent recovery stages.

### 3.2. Relationship between Extract Antioxidant Properties

**Figure 1:**
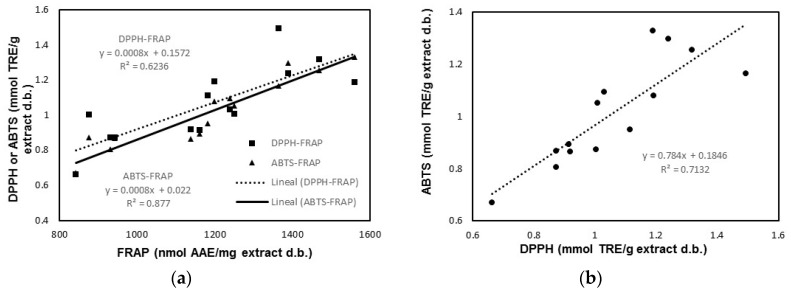
Linear relationships between FRAP and DPPH (2,2-diphenyl-1-picrylhydrazyl) and ABTS (2,2-azinobis-3-ethylbenzothiazoline-6-sulfonic acid) antioxidant activities (**a**) and between DPPH and ABTS antioxidant activities (**b**).

Antioxidants may respond in a different manner to different radical or oxidant sources [18]. For this reason, three methods based on different reaction mechanisms were used to determine the antioxidant activity of walnut leaf extracts and the relationship between them was analyzed. A regression analysis was performed between the values of the FRAP, DPPH, and ABTS antioxidant activities for the experiments in Table 2. As shown in Figure 1, quite good positive linear relationships were found between the three methods used to measure the antioxidant activity, which suggests that only one method could be used in practice to provide reliable information on the antioxidant properties of walnut leaf extracts.

### 3.3. Optimization of the Extraction Conditions by a Box-Behnken Design

In order to determine the best extraction conditions for the extraction of phenolic compounds from walnut leaves, a Box-Behnken experimental design was applied. The independent variables selected were temperature, time, and ethanol concentration. The extraction conditions together with the experimental results obtained for the dependent variables: Extraction yield (*Y*_1_), FRAP (*Y*_2_), DPPH (*Y*_3_), and ABTS (*Y*_4_) antioxidant activities are shown in Table 2, that also shows the good agreement between experimental and predicted values. The fitting model (Equation (1)) was found appropriate to represent all the responses analyzed (*p* < 0.05). Table 4 shows the significant regression coefficients together with the statistical parameters used to evaluate the fitting results. For all the responses, the quadratic effect of %EtOH (in negative mode) was the most significant, followed by the linear effect of temperature (in positive mode), and the effects on which extraction time was involved were the least significant or not significant at all. Equations (2)–(5) show the dependence of the responses *Y*_1_ to *Y*_4_ on the significant independent variables, double interactions, and quadratic effects:

(2) $$Y_{1}\;(\%)\;=\;26.28\;+\;3.024\;x_{1}^{*}+0.905\;x_{2}^{*}-1.444\;x_{3}^{*}-0.865\;x_{1}^{*}x_{3}^{*}-3.973\;x_{3}^{*2}$$

(3) $$Y_{2}\;(\mathrm{nmol}\;\mathrm{AAE}/\mathrm{mg}\;\mathrm{extract}\;d.b.)\;=\;1395.25\;+\;13.25\;x_{1}^{*}-354.50\;x_{3}^{*2}\;$$

(4) $$Y_{3}\;(\mathrm{mmol}\;\mathrm{TRE}/g\;\mathrm{extract}\;d.b.)\;=\;1.223\;+\;0.173\;x_{1}^{*}-0.093\;x_{1}^{*}x_{3}^{*}-0.291\;x_{3}^{*2}$$

(5) $$Y_{4}\;(\mathrm{mmol}\;\mathrm{TRE}/g\;\mathrm{extract}\;d.b.)\;=\;1.315\;+\;0.143\;x_{1}^{*}-0.083\;x_{1}^{*2}-0.125\;x_{2}^{*2}-0.315\;x_{3}^{*2}$$

**Figure 2:**
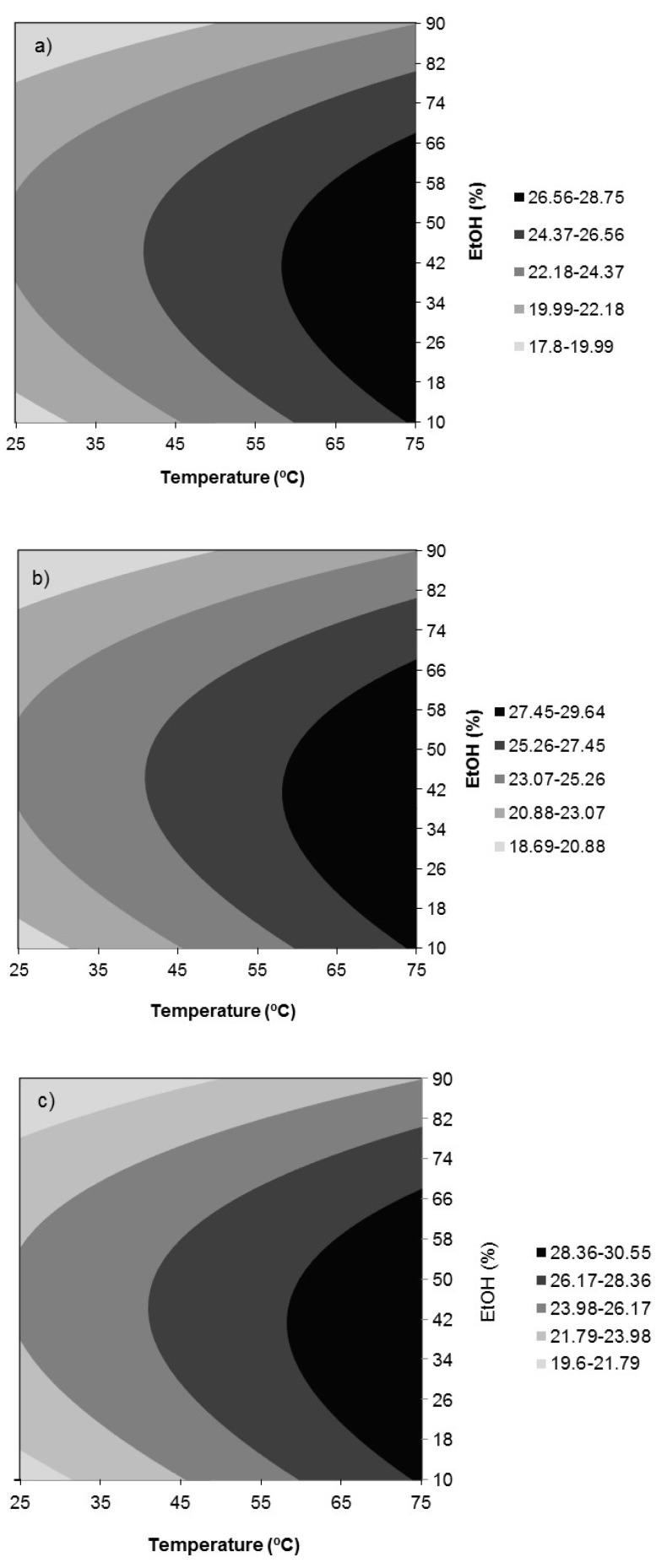
Response surfaces for extraction yield (*Y*_1_, %) in function of temperature and ethanol concentration for extraction times of (**a**) 30, (**b**) 75, and (**c**) 120 min.

Figure 2a–c show the response surfaces for the extraction yield (*Y*_1_) in the function of temperature and ethanol concentration for extraction times of 30 (a), 75 (b), and 120 min (c). Extraction yield varied between 17.80% and 30.55% depending on the extraction conditions. Regarding the influence of the linear effects, temperature and time were significant in the positive mode, whereas %EtOH in the negative mode. Only the quadratic effect of %EtOH and the double interaction temperature *x* %EtOH had significant influences on the response, both in the negative mode. The highest extraction yield in the ranges essayed of 30.55% was attained at the highest temperature and extraction time essayed, 75 °C and 120 min, and at an intermediate ethanol concentration, 38%. 

**Figure 3:**
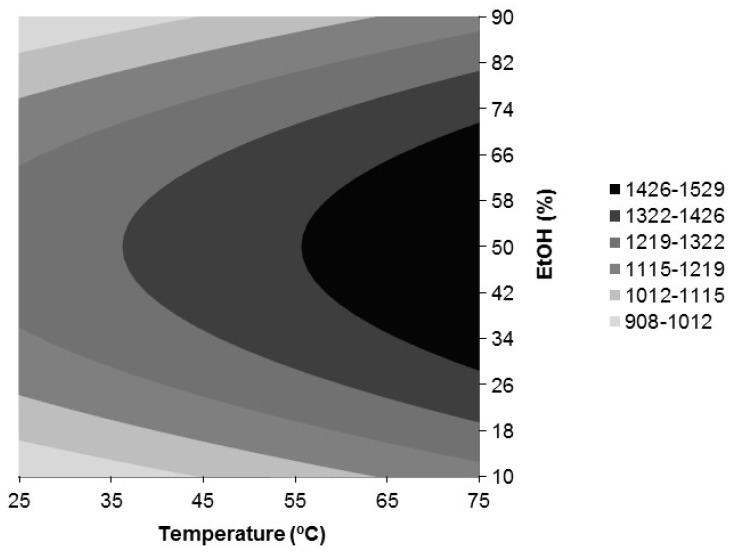
Response surfaces for FRAP antioxidant activity (*Y*_2_, nmol AAE/mg extract d.b.) in the function of temperature and ethanol concentration.

**Figure 4:**
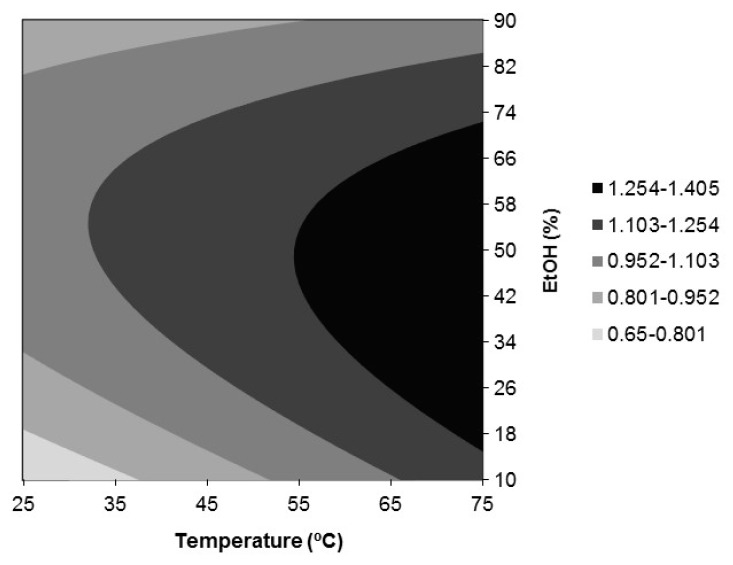
Response surfaces for DPPH antioxidant activity (*Y*_3_, mmol TRE/g extract d.b.) in the function of temperature and ethanol concentration.

**Figure 5:**
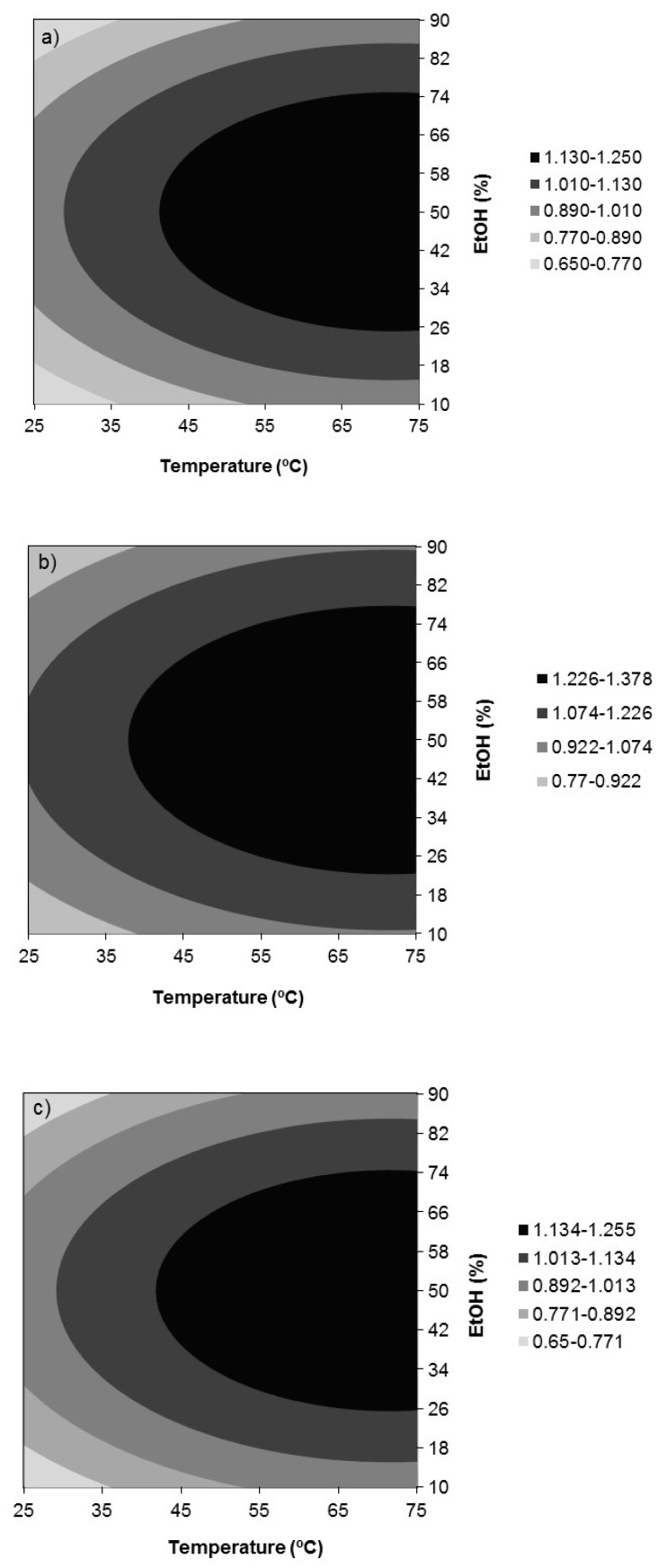
Response surfaces for ABTS antioxidant activity (*Y*_4_, mmol TRE/g extract d.b.) in the function of temperature and ethanol concentration for extraction times of (**a**) 30, (**b**) 75, and (**c**) 120 min.

Figure 3, Figure 4 and Figure 5 show, respectively, the response surfaces for FRAP (*Y*_2_), DPPH (*Y*_3_), and ABTS (*Y*_4_) antioxidant activities in function of temperature and ethanol concentration. All show a similar trend with respect to the influence of temperature and %EtOH, showing the presence of a maximum located in the vicinity of the design space for FRAP and DPPH and in the case of ABTS within it. Additionally, the responses for FRAP and DPPH were independent of time. FRAP ranged between 908 and 1529 nmol AAE/mg extract d.b. (Figure 3) and depended only on the negative quadratic effect of % EtOH and the linear effect of temperature in positive mode, reaching the highest value in the range assayed, at 75 °C and 50% ethanol. With respect to DPPH, it depended additionally on the interaction temperature *x* %EtOH in the negative mode, and a value of 1.40 mmol TRE/g extract d.b. was reached at 75 °C and 44% ethanol (Figure 4). ABTS depended on the quadratic effects of %EtOH, time and temperature, in this order of importance, all in the negative mode, and on the positive linear effect of temperature. For ABTS, a maximum located in the region studied, 1.38 mmol TRE/g extract d.b., was reached at 71.3 °C, 75 min, and 50% ethanol (Figure 5).

In summary, solvent concentration, temperature, and extraction time, in this order, are important factors influencing the extraction of phenolic compounds from walnut leaves. Both extraction yield and extract antioxidant activity depended significantly on the % ethanol used and aqueous solutions in the range of 38% to 50% ethanol were found to be superior to the more concentrated ones in any of the solvents, behavior that might be explained by the “likes dissolves like” principle [19]. High ethanol concentrations dissolve more lipophilic compounds, whereas higher proportions of hydrophilic compounds are extracted at low ethanol concentrations [20]. Otherwise, extraction yield increases when decreasing %EtOH up to 38%, whereas extract antioxidant capacity diminishes, which can be explained due to the solubilization of other types of compounds such as proteins and polysaccharides that impair selectivity [21]. According to these results, the ethanol concentration selected as the optimum was 50%. Concerning extraction temperature, a temperature rise of up to 75 °C increased the extraction yield as the extraction process was favored by temperature as the solubility of phenolic compounds and the mass transfer rate were enhanced [22]. Moreover, FRAP, DPPH, and ABTS antioxidant activity of the extracts also increased with increasing extraction temperature to 75 °C. However, greater extraction temperatures were not recommended as oxidation, epimerization, and degradation of phenolic compounds was promoted [17] with the consequent decrease of the extract antioxidant activity. Therefore, 75 °C was selected as the optimum temperature. Finally, 120 min was the selected time to favor the extraction yield, as its influence on antioxidant properties was almost negligible. Longer extraction times were not proposed since the effects were the same as those previously indicated for high temperatures [17].

In brief, the optimal conditions for the extraction of phenolic compounds from *Juglans major 209 x Juglans regia* leaves selected by analyzing together the response surfaces for all the dependent variables (Figure 2, Figure 3, Figure 4 and Figure 5) were those corresponding to Experiment 12 (Table 2): 75 °C, 120 min, and 50% ethanol. Under these conditions, the predicted responses were: *Y*_1, pred_ = 30.21%, *Y*_2, pred_ = 1529 nmol AAE/mg extract d.b., *Y*_3, pred_ = 1.396 mmol TRE/g extract d.b. and *Y*_4, pred_ = 1.25 mmol TRE/g extract d.b. These values were equal or very close to those corresponding to the model optimum for each response that were *Y*_1,pred_ = 30.55% at 75 °C, 120 min, and 38% EtOH, *Y*_2,pred_ = 1529 nmol AAE/mg extract d.b. at 75 °C and 50% EtOH, *Y*_3,pred_ = 1.40 mmol TRE/g extract d.b. at 75 °C and 44% EtOH and *Y*_4,pred_ = 1.38 mmol TRE/g extract d.b. at 71.3 °C, 75 min and 50% EtOH. Moreover, these extraction conditions were close to those found by Vieira et al. [7] as the global optimum conditions for the extraction of some of the main phenolic compounds found in this work, namely, 3-*O*-caffeoylquinic acid and quercetin 3-*O*-glucoside, from walnut (*Juglans regia*) leaves by maceration with aqueous ethanol (61.3 °C, 112.5 min, and 50.4% ethanol). 50% ethanol also led to higher antioxidant activities for walnut (*Juglans regia*) green husk extracts than pure water and ethanol, although the highest extraction yield was obtained with water [23].

### 3.4. Characterization of the Optimum Walnut Leaf Extract

**Figure 6:**
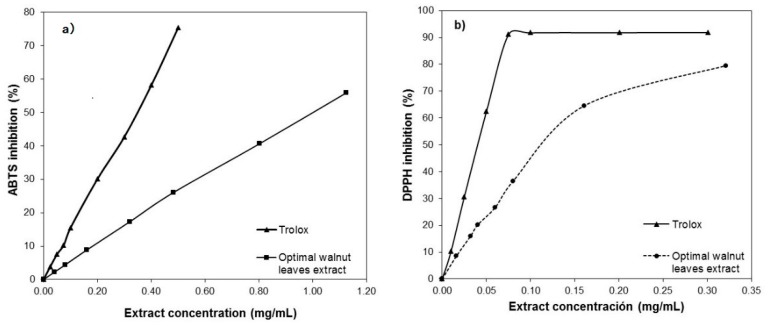
Percentage of ABTS (**a**) and DPPH (**b**) radical inhibition against extract concentration for the walnut leaf extract selected as the optimum with Trolox as reference.

Both the experimental values and those predicted by the models (Equations (2)–(5), Table 4) for the extraction yield and antioxidant properties of the walnut leaf extract selected as the optimum are presented in Table 2 (Experiment 12). Additionally, Figure 6a,b show the ABTS and DPPH radical inhibition percentages, respectively, versus extract concentration for this walnut extract and for Trolox, the synthetic antioxidant used as a reference. The corresponding EC_50_ values were 0.985 and 0.11 mg/mL, for ABTS and DDPH, respectively. The DPPH EC_50_ value obtained in this work was lower (higher antioxidant activity) than those found for extracts obtained from leaves of different *Juglans regia* cultivars using other solvent systems: Aqueous extracts (0.151–0.202 mg/mL) [1], methanolic extracts (0.199 mg/mL), and petroleum ether extract (2.921 mg/mL) [3]. However, even lower EC_50_ values were obtained for methanolic (65.91 µg/mL) and decoction (78.97 µg/mL) extracts from certain walnut cultivars [4]. The DPPH EC_50_ value obtained in this work for the optimum walnut leaf extract was also significantly lower than that for *Juglans regia* green husk extracts, 0.33 mg/mL for 50% aqueous ethanol extracts [23], and 0.41 for methanolic extracts [3], and also slightly lower than that for the methanolic extract from walnut seeds (0.14 mg/mL) [3]. With respect to extraction yield it was slightly higher than that reported by Carvalho et al. [3] for the methanolic extract, 27.7%, and significantly higher than that of the petroleum ether ones, 1.1%.

**Figure 7:**
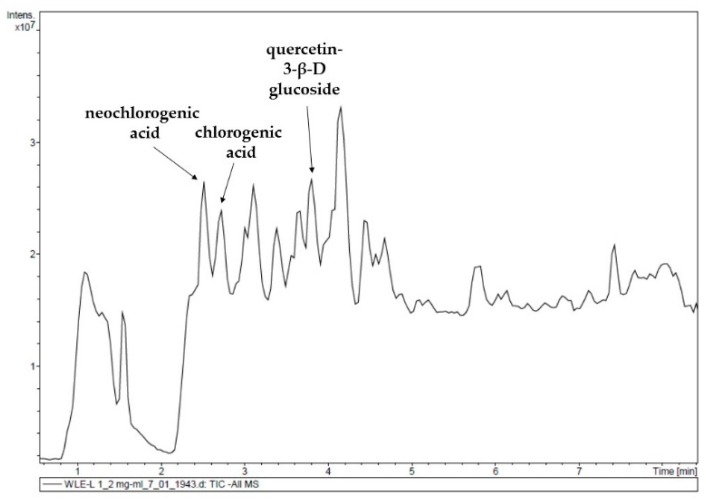
UPLC chromatogram of the walnut leaf extract selected as the optimum (*T* = 75 °C, *t* = 120 min, EtOH concentration = 50%, S/L ratio = 1/10 (g/mL).

Finally, to identify and quantify the compounds responsible for the antioxidant activity of the optimum *Juglans major 209 x Juglans regia* leaf extract, it was analyzed by UPLC/ESI-QTOF-MS. Table 5 and Figure 7 show the phenolic profile determined. Fifteen phenolic compounds were identified and quantified, with the flavonoid quercetin 3-*β*-D-glucoside being the major compound, representing 49.35% of the total phenolic composition, followed by chlorogenic acid (29.16%), and neochlorogenic acid (18.34%). Both quercetin 3-*β*-D-glucoside and chlorogenic acid were among the main phenolic compounds found in *Juglans regia* leaf extracts from some Spanish and Portuguese cultivars [1,4,7] that also presented significant quantities of other flavonoids such as quercetin 3-*O*-galactoside, quercetin *O*-pentoside, or quercetin *O*-xyloside [1,4,5,7] not identified in this work. However, the outstanding presence of neochlorogenic acid in the *Juglans major 209 x Juglans regia* leaf extracts, also reported by Pereira et al. [1], makes the main difference with the *Juglans regia* ones.

Concerning the interest of the main phenolic compounds identified, quercetin 3-*β*-D glucoside has been attracting increasing research interest. It is widely distributed in fruits, vegetables, and cereals and exhibits a broad number of chemoprotective effects both in vitro and in vivo, against oxidative stress, cancer, cardiovascular disorders, diabetes, and allergic reactions [24]. Chlorogenic acids are also present in many vegetables and fruits and some health benefits have been associated with them such as the prevention of cardiovascular diseases and types 2 diabetes [25] and also antioxidants, anticancer, anti-inflammatory, and immunomodulatory properties have been shown [26]. The importance of these phenolic compounds for various industrial applications demonstrate the interest of the valorization of *Juglans major 209 x Juglans regia* leaves. 

## 4. Conclusions

Phenolic compounds such as quercetin 3-*β*-D-glucoside (4.92 mg/g), neochlorogenic acid (2.91 mg/g), and chlorogenic acid (1.83 mg/g) have been recovered from walnut (*Juglans major 209 x Juglans regia*) leaves by maceration in aqueous ethanol solutions under optimized conditions, 75 °C, 120 min, and 50% aqueous ethanol, selected based on extraction yield and extract antioxidant properties. The experimental values for the optimal extract properties were: Extraction yield, 30.17%; FRAP, 1468 nmol AAE/mg extract d.b.; DPPH, 1.318 mmol TRE/g extract d.b.; DPPH EC_50_, 0.11 mg/mL; ABTS, 1.256 mmol TRE/g extract d.b. and ABTS EC_50_, 0.985 mg/mL. Therefore, the present work proposes a valorization way of walnut leaves as a source of valuable compounds for various industrial applications based on their antioxidant properties.

## Figures and Tables

**Table 1:** Influence of the solid/liquid ratio on extraction yield and ferric reducing antioxidant power (FRAP) antioxidant activity of walnut leaf extracts (50 °C, 60 min, and 50% aqueous ethanol).

Exp.	S/L Ratio (g/mL)	Extraction Yield (%)	FRAP (nmol AAE/mg Extract)
A	1/5	25.82 ± 0.25 ^a^	1246 ± 48 ^a^
B	1/7.5	27.04 ± 0.42 ^b^	1350 ± 53 ^b^
C	1/10	27.86 ± 0.04 ^b^	1512 ± 61 ^c^

Values are presented as mean ± standard deviation. ^a–c^ In each column, values with different letters are significantly different (*p <* 0.05).

**Table 2:** Box-Benkhen experimental design with the experimental and predicted values of the responses.

Exp	$x_{1}^{*}$	$x_{2}^{*}$	$x_{3}^{*}$	*Y*_1 exp_	*Y*_1 pred_	*Y*_2 exp_	*Y*_2 pred_	*Y*_3 exp_	*Y*_3 pred_	*Y*_4 exp_	*Y*_4 pred_
1	−1	−1	0	21.03 ± 0.89	22.35	1181 ± 39	1262	1.114 ± 0.026	1.050	0.951 ± 0.026	0.964
2	−1	0	−1	20.35 ± 0.18	19.86	842 ± 46	908	0.663 ± 0.044	0.666	0.671 ± 0.027	0.774
3	−1	0	1	19.15 ± 2.13	18.71	930 ± 47	908	0.873 ± 0.013	0.852	0.807 ± 0.017	0.774
4	−1	1	0	23.89 ± 0.28	24.16	1250 ± 58	1262	1.008 ± 0.159	1.050	1.053 ± 0.049	0.964
5	0	−1	−1	23.13 ± 0.27	22.85	1160 ± 37	1041	0.913 ± 0.024	0.932	0.893 ± 0.041	0.875
6	0	−1	1	20.03 ± 1.16	19.96	1138 ± 17	1041	0.919 ± 0.039	0.932	0.866 ± 0.050	0.875
7	0	1	−1	24.52 ± 0.11	24.66	943 ± 65	1041	0.873 ± 0.037	0.932	0.869 ± 0.030	0.875
8	0	1	1	21.93 ± 1.76	21.77	876 ± 18	1041	1.003 ± 0.036	0.932	0.875 ± 0.028	0.875
9	1	−1	0	29.08 ± 1.43	28.40	1364 ± 42	1529	1.493 ± 0.106	1.396	1.166 ± 0.047	1.250
10	1	0	−1	27.01 ± 0.30	27.64	1199 ± 50	1174	1.193 ± 0.021	1.198	1.089 ± 0.035	1.060
11	1	0	1	22.35 ± 0.88	23.02	1238 ± 32	1174	1.031 ± 0.023	1.012	1.095 ± 0.032	1.060
12	1	1	0	30.17 ± 0.09	30.21	1468 ± 20	1529	1.318 ± 0.049	1.396	1.256 ± 0.017	1.250
13	0	0	0	25.85 ± 0.05	26.28	1389 ± 78	1395	1.239 ± 0.043	1.223	1.299 ± 0.049	1.315
14	0	0	0	27.19 ± 0.51	26.28	1560 ± 49	1395	1.190 ± 0.031	1.223	1.330 ± 0.032	1.315
**Independent Variables**						**Levels**					
					−1	0	+1				
*x*_1_, Temperature (°C)					25	50	75				
*x*_2_, Time (min)					30	75	120				
*x*_3_, % EtOH					10	50	90				

Experimental values are presented as mean ± standard deviation. *Y*_1_, extraction yield (g extract/100 g leaves d.b.); antioxidant activity: *Y*_2_, FRAP (nmol AAE/mg extract d.b.); *Y*_3_, DPPH (mmol TRE/g extract d.b.); *Y*_4_, ABTS (mmol TRE/g extract d.b.); $x_{1}^{*}$, codified temperature; $x_{2}^{*}$, codified time; $x_{3}^{*}$, codified ethanol concentration.

**Table 3:** Calibration curves for the standard compounds analyzed by ultra-performance liquid chromatography coupled with electrospray ionization and time-of-flight mass spectrometry (UPLC/ESI-QTOF-MS).

Compound	Linear Range (mg/L)	Calibration Curve	*R* ^2^
(–)-Gallocatechin	1–200	*y* = 8068*x* + 17,932	0.9941
Catechin hydrate	1–200	*y* = 15,461*x* + 52,943	0.9958
Chlorogenic acid	1–1000	*y* = 8181*x* + 22,563	0.9936
Ellagic acid	1–1000	*y* = 9647*x* + 38,987	0.9959
Epicatechin	1–1000	*y* = 9780*x* + 17,102	0.9976
Ferulic acid	1–200	*y* = 3455*x* + 22,085	0.9936
Gallic acid	1–1000	*y* = 3814*x* + 4250	0.9977
Isorharmnetin	1–200	*y* = 61,453*x* + 78,552	0.9915
Kaempferol	1–200	*y* = 61,712*x* + 75,935	0.9923
Neochlorogenic acid	1–200	*y* = 10,675*x* + 15,125	0.9989
*p*-Coumaric acid	1–200	*y* = 4442*x* + 4535	0.9972
Procyanidin B2	1–200	*y* = 4014*x* + 7252	0.9925
Quercetin	1–1000	*y* = 45,006*x* + 111,541	0.9922
Quercetin 3-*β*-d-glucoside	1–1000	*y* = 13,239*x* + 42,498	0.9836
Taxifolin	1–200	*y* = 21,398*x* + 28,956	0.9903

**Table 4:** Coefficients of the models (Equation (1)) and statistical parameters.

	*Y*_1_			*Y*_2_			*Y*_3_			*Y*_4_		
	Coeff.	SE	*p*	Coeff	SE	*p*	Coeff.	SE	*p*	Coeff.	SE	*p*
*a*_0_	26.281	0.274	0.000	1395.250	40.672	0.000	1.223	0.019	0.000	1.315	0.028	0.000
*a*_1_	3.024	0.274	0.000	13.25	40.672	0.006	0.173	0.019	0.000	0.143	0.019	0.000
*a*_2_	0.905	0.274	0.008	-	-	NS	-	-	NS	-	-	NS
*a*_3_	−1.444	0.274	0.000	-	-	NS	-	-	NS	-	-	NS
*a*_12_	-	-	NS	-	-	NS	-	-	NS	-	-	NS
*a*_13_	−0.865	0.387	0.049	-	-	NS	−0.093	0.027	0.005	-	-	NS
*a*_23_	-	-	NS	-	-	NS	-	-	NS	-	-	NS
*a*_11_	-	-	NS	-	-	NS	-	-	NS	−0.083	0.028	0.012
*a*_22_	-	-	NS	-	-	NS	-	-	NS	−0.125	0.028	0.001
*a*_33_	−3.973	0.387	0.000	−354.500	57.519	0.000	−0.291	0.027	0.000	−0.315	0.028	0.000
*R* ^2^	0.964			0.789			0.947			0.951		
*R*^2^corr.	0.947			0.757			0.934			0.933		
SE	0.774			115.038			0.053			0.055		
*p*	0.000			0.000			0.000			0.000		

NS: Non-significant for a 95% confidence level; SE: Standard error; *p*: Probability; *R*^2^: Regression coefficient; *R*^2^_c_: Corrected regression coefficient; *a*_0_: Scaling constant; *a*_i_: Linear coefficients; *a*_ij_: Interaction coefficients; *a*_ii_: Quadratic coefficients; *Y*_1_, extraction yield (g extract/100 g leaves d.b.); antioxidant activity: *Y*_2_, FRAP (nmol AAE/mg extract d.b.); *Y*_3_, DPPH (mmol TRE/g extract d.b.); *Y*_4_, ABTS (mmol TRE/g extract d.b.).

**Table 5:** Identified compounds in the optimized walnut leaf extract by UPLC/ESI-QTOF-MS (*T* = 75 °C, *t* = 120 min, EtOH concentration = 50%, S/L ratio = 1/10 g/mL).

Compound	Retention Time (min)	*m/z*	ng/mg Extract d.b.	mg/100 g Leaves d.b.
(–)-Gallocatechin	2.4	305.06	14.17	0.45
Catechin hydrate	2.6	289.07	55.83	1.78
Chlorogenic acid	2.7	353.09	5737.50	183.14
Ellagic Acid	4	300.99	68.92	2.20
Epicatechin	2.8	289.07	12.08	0.39
Ferulic acid	3.6	193.04	158.58	5.06
Gallic acid	2.3	169.01	432.58	13.81
Isorharmnetin	5.4	315.05	3.17	0.10
Kaempferol	5.3	285.04	14.25	0.45
Neochlorogenic acid	2.5	353.08	9125.00	291.27
*p*-Coumaric ccid	3.6	163.04	10.83	0.35
Procyanidin B2	2.6	577.14	45.50	1.45
Quercetin	4.8	301.04	163.50	5.22
Quercetin 3-*β*-d-glucoside	3.8	463.09	15,441.67	492.90
Taxifolin	3.4	303.05	4.50	0.14
